# Topical nifedipine for post-haemorrhoidectomy pain relief: randomized, prospective, double-blind trial protocol

**DOI:** 10.1093/bjsopen/zrad095

**Published:** 2023-09-21

**Authors:** Christopher J Steen, Raymond J Yap, Mohammad Asghari-Jafarabadi, Adam Sutton, Martin Chin, Peter Carne, Stephen W Bell, Paul J McMurrick

**Affiliations:** Department of Surgery, Cabrini Monash University, Melbourne, Victoria, Australia; Department of Surgery, Cabrini Monash University, Melbourne, Victoria, Australia; Department of Surgery, Cabrini Monash University, Melbourne, Victoria, Australia; Department of Surgery, Cabrini Monash University, Melbourne, Victoria, Australia; Department of Surgery, Cabrini Monash University, Melbourne, Victoria, Australia; Department of Surgery, Cabrini Monash University, Melbourne, Victoria, Australia; Department of Surgery, Cabrini Monash University, Melbourne, Victoria, Australia; Department of Surgery, Cabrini Monash University, Melbourne, Victoria, Australia

## Introduction

Haemorrhoids are a common condition worldwide and are thought to be a result of prolapsed anal cushions that have undergone pathological changes^1^. Haemorrhoids can be either external or internal, with the latter graded on a scale of grade I–IV^2^. A number of non-operative and operative strategies have been implemented to treat symptomatic haemorrhoids, but, for larger grade III–IV haemorrhoids, a surgical haemorrhoidectomy remains the gold standard^2–4^. However, haemorrhoidectomy is also associated with adverse effects, with postoperative pain being the most common^2–4^.

Post-haemorrhoidectomy pain is thought to be related to spasm of the internal anal sphincter^4–6^, but other causes may include local nerve-end damage, creation of an anal fissure, or suture placement below the dentate line^4,6–8^. Numerous modalities of pain management have been suggested, with multiple studies looking at the use of topical pain relief. The studies have shown promising, albeit mixed, results, examining the use of topical interventions like lignocaine, nitroglycerin, metronidazole, and diltiazem^2,6,7,9–13^.

Calcium channel blockers (CCBs), available in topical preparations, are thought to decrease internal sphincter spasm due to blockage of myocytes and subsequent relaxation of smooth muscles^6,14^. One such CCB, diltiazem, has shown success in multiple RCTs in significantly reducing post-haemorrhoidectomy pain and likely reducing overall opioid use amongst patients^9–13^. Another CCB, nifedipine, has been less documented in the literature with respect to its topical use in relieving post-haemorrhoidectomy pain. One RCT was able to show a significant reduction in pain relief post-haemorrhoidectomy when topical nifedipine was combined with lignocaine *versus* a control group of lignocaine alone^4^. Additionally, multiple studies examining chronic anal fissures suggest that nifedipine appears to have more effective healing rates than diltiazem^15,16^. Thus, given the lack of literature on the use of topical nifedipine in post-haemorrhoidectomy patients, and its reported success in healing anal fissures, the planned study seeks to establish if nifedipine alone, like diltiazem, *versus* a topical placebo, can reduce postoperative pain, as well as limit opioid use.

## Methods

The primary study hypothesis is that topical nifedipine (0.5 per cent) ointment, compared with topical placebo alone, leads to a significant reduction in pain experienced by patients post-haemorrhoidectomy. The secondary study hypothesis is that topical nifedipine ointment leads to less use of rescue oral opioid analgesia by patients, post-haemorrhoidectomy, who are on a standardized postoperative analgesia regimen, with no significant differences in adverse effects between those receiving the topical nifedipine ointment intervention *versus* those receiving the placebo ointment.

### Study design and selection criteria

**Fig. 1:**
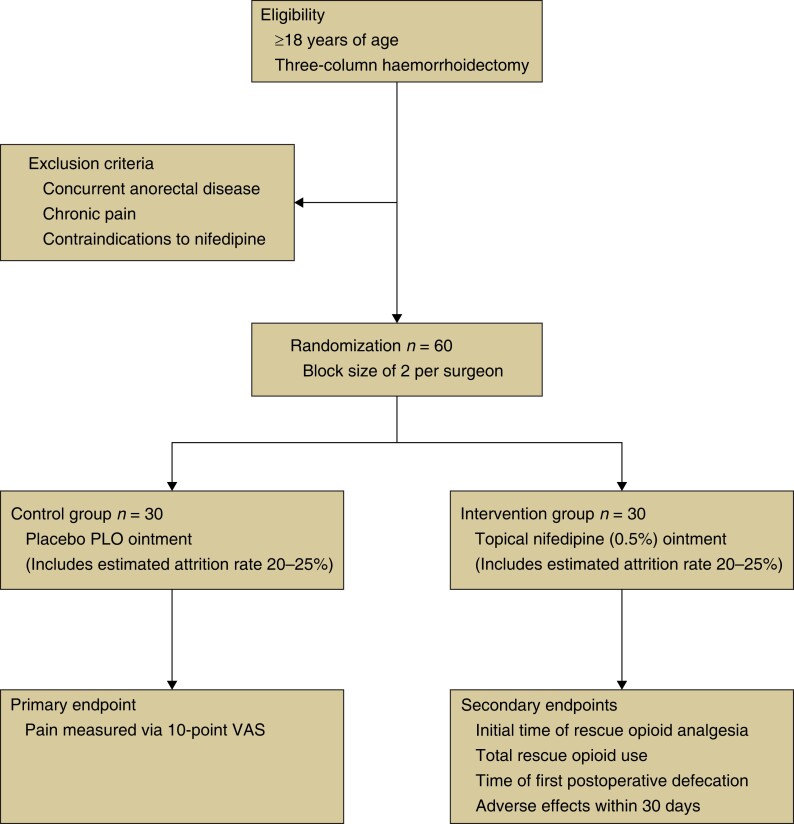
Trial design PLO, pluronic lecithin organogel; VAS, visual analogue scale.

A single-centre, randomized, prospective, double-blind placebo trial will be conducted comparing the efficacy of topical nifedipine ointment *versus* a topical placebo ointment alone in reducing post-haemorrhoidectomy pain. Secondary outcomes will include rescue oral opioid analgesia use and adverse effects (*Fig. 1*). The trial began in July 2023 and aims to finish recruitment by July 2024.

Patients over the age of 18 years who are scheduled to undergo three-column haemorrhoidectomy, using a closed technique, will be considered for inclusion and will be recruited by surgeons during either their initial consultation for haemorrhoidectomy surgery or on the day of planned surgery. Exclusion criteria will be patients who: are younger than 18 years; are pregnant or breastfeeding; have concurrent non-haemorrhoidal anorectal disease; have a known allergy to nifedipine; have co-morbidities or take medications that are a contraindication to nifedipine use; have impaired renal function where parecoxib is contraindicated; have a chronic pain condition requiring ongoing regular analgesia; cannot provide informed consent; and cannot have a general anaesthetic.

For each included patient, demographics and clinical data will be recorded as reported in the *Supplementary material*.

### Group allocation

After written informed consent, patients will be randomized to either the intervention group (topical nifedipine ointment) or the control group (placebo ointment) by computer randomization. This will be achieved through the use of a random sequence generated by the study statistician using a package created using MS Excel 2013 software. The patients will be allocated using a randomized block procedure, with a block size of two. Surgeons will be blinded to this allocation.

### Standardized analgesia regimen

All patients will receive a general anaesthetic along with intravenous paracetamol (1 g), alfentanil (1 mg), oxycodone (0.05–0.1 mg/kg), and parecoxib (40 mg). After surgery patients will receive recovery analgesia consisting of intravenous oxycodone (2 mg), as required, with a maximum of 10 mg available. Also, local anaesthetic consisting of 20 ml bupivacaine (0.5 per cent) with adrenaline (1 : 200 000) will be used for local wound infiltration.

Upon discharge, patients will be provided with a prescription for regular oral paracetamol (1 g four times per day) and celecoxib (100 mg twice per day) for 5 days (to start 18 h post-parecoxib). They will also receive rescue oxycodone (5 mg every 4 h, with a maximum daily dose of 25 mg), with 20 tablets (100 mg), in total, available.

### Intervention and placebo

Patients will receive a supply of anorectal ointment that is de-identified and randomized, that is either the intervention nifedipine ointment or the placebo ointment, thus blinding the patients appropriately. The intervention ointment will consist of a jar containing 30 g of nifedipine (0.5 per cent) in pluronic lecithin organogel (PLO), a common compounding medium for topical medications, with the control group receiving a placebo of PLO only. All patients will be instructed to apply a pea-sized amount of their ointment, using a glove, 1–1.5 cm into the anus circumferentially, as well as to the external anus. This will be done twice a day for 4 weeks.

### Outcome measures

As stated, the primary outcome measured will be post-haemorrhoidectomy pain experienced by subjects. This will be measured using a 10-point standardized and validated visual analogue scale (‘VAS’). Scores will range from 0 (no pain at all) to 10 (worst pain imaginable). Pain scores will be recorded at the following time points: before surgery (baseline); at recovery; at 4 h (discharge); daily (week 1); and at 2, 3, and 4 weeks.

Secondary outcomes measured will include rescue analgesic use and adverse effects (*Supplementary material*).

### Statistics and sample size

The statistical plan and intention-to-treat analyses are detailed in the *Supplementary material*. Compared with similar studies, pre-trial calculations established a required sample size of 48 (24 patients per group), based on α = 0.05 and power = 0.9, based on the primary outcome (pain). Estimating an attrition rate of 20–25 per cent, a sample size of 60 (30 per group) was chosen. The sample size was calculated utilizing STATA 17 (StataCorp, College Station, TX, USA) considering a two-tailed test.

### Ethics

An ethics application has been submitted to the local Cabrini Monash University Human Research Ethics Committee. The trial is registered in the Australia New Zealand Clinical Trials Registry (ANZCTR 12623000514606p). Recruitment started in July 2023 and is expected to finish in July 2024.

## Discussion

Post-haemorrhoidectomy pain has a pathophysiology that is proposed to be similar to non-healing chronic anal fissures. Given the success of topical nifedipine in healing anal fissures, the authors present a protocol for a single-centre, double-blind, placebo RCT assessing the use of topical nifedipine in haemorrhoidectomy patients, with post-operative pain as the primary outcome.

The literature supports the use of topical CCB diltiazem to relieve post-haemorrhoidectomy pain; however, to the authors’ knowledge, the planned study will be the first to examine the use of nifedipine alone in this cohort. Additionally, the authors aim to better establish guidelines on the administration, dosing, and length of time for topical nifedipine use in this cohort. Furthermore, the planned study will investigate if there is a difference in the use of rescue oral opioid analgesia and any reported adverse effects between the groups.

## Supplementary Material

zrad095_Supplementary_Data

## Data Availability

All relevant data will be published upon completion of the trial.
