# Supplementary material for: Topical nifedipine for post-haemorrhoidectomy pain relief: randomized, prospective, double-blind trial protocol
Source: BJS Open. 2023 Sep 21;7(5):zrad095. doi: 10.1093/bjsopen/zrad095 (PMC10516616; doi:10.1093/bjsopen/zrad095)
Supplement: zrad095_Supplementary_Data [file zrad095_supplementary_data.docx]

**Topical nifedipine for post haemorrhoidectomy pain relief: a randomised, prospective, double-blind placebo trial protocol**

Christopher J Steen, Raymond J Yap, Mohammad Asghari-Jafarabadi, Adam Sutton, Martin Chin, Peter Carne, Stephen W Bell, Paul J McMurrick

^1^Cabrini Monash University, Department of Surgery, Melbourne, Australia

Corresponding Author:

Dr Christopher J Steen

Suite 20, 181 Wattletree Rd

Malvern, VIC 3144

Australia

Orchid ID 0000-0001-5057-6436

**Supplementary Materials - Index**

| **Supplementary Methods** |  |
| --- | --- |
| Primary outcome measures | *page 2* |
| Secondary outcome measures | *page 2* |
| Demographics | *page 2* |
| Data collection | *page 3* |
| Statistical analysis | *page 3* |

**Supplementary Methods**

*Measures*

*Primary outcome measures*

As stated, the primary outcome measured will be post haemorrhoidectomy pain experienced by subjects. This will be measured using a 10-point standardised and validated visual analogue scale (VAS). Scores will range from zero (no pain at all) to ten (worst pain imaginable). Pain scores will be recorded at the following timepoints:

1. pre-operatively (baseline)
2. recovery
3. 4 hours (discharge)
4. daily (week 1)
5. 2, 3 and 4 weeks

*Secondary outcome measures*

Secondary outcomes that will be measured include the following:

1. initial time of rescue oxycodone analgesia usage
2. total rescue oxycodone usage
3. time to first defecation
4. adverse events within the first 30 days:
   - readmission
   - reoperation
   - urinary retention requiring catherisation
   - perianal bleeding
   - perianal swelling or oedema
   - faecal incontinence
   - tenesmus
   - pain on defecation
   - headache
   - light-headedness or dizziness
   - peripheral oedema

*Demographics*

Demographics of each patient participating in the trial will be collected and will include, but not be limited by, the following:

1. age
2. sex/gender
3. ethnicity
4. BMI
5. relevant comorbidities
6. ASA grade
7. haemorrhoid degree (largest)

*Data collection*

Prior to and following haemorrhoidectomy, nursing staff will collect inpatient pain scores by paper survey. All subsequent pain scores and secondary outcomes will be collected via online patient surveys that will be sent to the patients by email or SMS. Data will be de-identified and recorded in a secure, password protected database at Cabrini Hospital. Data will be kept for a period of 5 years to allow for publication and dissemination of results.

*Statistical analyses*

The statistical analysis for this study will be conducted using STATA 17 (StataCorp, College Station, Texas 77845 USA). To ensure the validity of our findings, we will first check the normality of the numeric variables using the Kolmogorov-Smirnov test, as well as descriptive measures of distribution such as skewness and kurtosis.

For presentation purposes, we will report mean (SD) for numeric normal variables, median (min-max) for non-normal variables, and frequency (percent) for categorical variables. To compare baseline measures and demographic variables between the two groups, we will use independent t-tests and Fisher-Freeman-Halton Exact tests where appropriate.

To assess the effect of the intervention, we will use two analysis of covariance (ANCOVA) models: model 1 will control for baseline measures (baseline adjusted), and model 2 will control for baseline measures and possible confounders (fully adjusted).

The study will utilize a two-way analysis of variance with repeated measures (RMANOVA) to evaluate the effect of the intervention, time, and their interactions on primary and secondary outcomes. The assumption of sphericity will be assessed using the Mauchly test and, to correct for any deviation from the assumption, Greenhouse-Geiser-based correction will be employed. Significant RMANOVA results will be followed by Sidak post-hoc tests.

We will also use an intention-to-treat (ITT) approach for all analyses, and will consider *p* values less than 0.05 to be statistically significant.
